# Physiological Chemistry

**Published:** 1895-09

**Authors:** John A. Miller, F. H. Mills


### PHYSIOLOGICAL CHEMISTRY.

CONDUCTED BY JOHN A. MILLER, PH. D., ASSISTED BY F. H. MILLS, M. D.

### SUGAR IN THE BLOOD AFTER BLEEDING.

F. SCHENCK (*Jour. Chem. Soc.*, 1895 ; *Pflüger's Archiv.*, 1894). Claude Bernard originally stated that loss of blood caused a rise of sugar in the residual blood. This was confirmed by v. Mering and also in the present research, where a new method was employed. The rise of sugar begins immediately after bleeding, but cannot be recognised a few hours after. The source of the sugar is apparently the liver, as the rise does not occur if the liver is cut off from the circulation. It is more marked when ammonium carbonate is given, this drug stimulating glycogenesis, and is diminished by glycenol, which inhibits the change of glycogen into sugar.

---

### PERCENTAGE OF IRON IN THE LIVER IN ANKYLOSTOMIASIS.

B. RAKE (*J. Pathol. and Bacteriol.*, 1894 ; *J. Chem. Soc.*, 1895). The percentage of iron in liver and spleen in five cases of disease, due to the presence of the worm *ankylostoma duodenale*, is given. The interest of such an investigation arises from the fact that the symptoms produced are very like those of pernicious anemia.

The average percentage of iron in the liver is less than in other diseases, and much less than in pernicious anemia ; thus : ankylostomiasis, 0.1 ; other diseases, 0.12 ; pernicious anemia, 0.7. The iron in the spleen is scarcely affected.

The intense anemia associated with the disease is simply due to loss of blood from the intestine, and is not caused by any toxic substance causing blood destruction in the liver.

---

### BACTERICIDAL ACTION OF LIGHT AND AIR.

R. F. D'ARCY and W. B. HARDY (*J. Physiol.*, 1894 ; *J. Chem. Soc.*, 1895). Marshall Ward has shown that the bactericidal power of light is a peculiar property of light of short wave length, and is at its maximum at the violet end of the blue. This is only manifested in the presence of oxygen, and Wnerster has shown that "active oxygen" is produced when evaporation takes place in direct sunlight. The present experiments show that when the spectrum of a powerful arc light is allowed to fall on a moist surface in the presence of a delicate indicator, oxidation occurs, and the action commences at the blue end of the green and continues through the blue, violet and ultra-violet regions.

---

### EFFECT OF SUNLIGHT ON TETANUS CULTURES.

F. F. WESBROOK (*J. Pathol. and Bacteriol.; J. Chem. Soc.*, 1895). The experiments confirm other observers in the conclusions that oxygen is a necessary factor in the destruction of bacteria by light. Without oxygen sunlight is powerless.

---

### CONTENTS OF THE HEALTHY STOMACH.

A. L. GILLESPIE (Rep. of Lab. R. C. P., Edin., 1894; *J. Chem. Soc.*, 1895). The following conclusions are drawn from the examination of the contents of the healthy stomach: (1) Free hydrochloric acid is secreted immediately the food enters; but this combines with proteïd, so that salivary action is not impeded during the first stage. The free acid does not appear in the stomach contents till from half an hour to two or three hours after the meal is taken; the time varies according to the composition of the meal. Hydrochloric acid combined with proteïd is less antiseptic than the free acid. The inorganic salts, especially the chlorides, and the total proteïds per cent. in solution fall during the progress of digestion. Albumin increases, albumoses remain stationary and peptone diminishes.

Physiological text-books are stated to be in error concerning the amount of acid. The following numbers are derived from the present observation: total acidity, from 0.108 to 0.36 per cent.; combined acidity, from 0.072 to 0.324 per cent.; free acidity, 0.018 to 0.09 per cent.

The causes of variation are the time after the ingestion of a meal and the character of the food taken. The free hydrochloric acid is seldom over 0.09 per cent. after a proteïd meal, but it may rise to 0.162 or 0.27 after a meal chiefly carbohydrate in nature.

---

### INFLUENCE OF FAT ON THE ASSIMILATION OF PROTEÏDS.

R. LAAS (*Zeit. Physiol. Chem.*, 1894; *J. Chem. Soc.*, 1895). The admixture of fat with proteïd food increases the nutritive value of the latter, more nitrogenous matter being assimilated. Fats, however, unlike carbohydrates, do not lessen the putrefactive changes in proteïds in the alimentary canal.

INFLUENCE OF CARBON DIOXIDE AND OXYGEN ON BLOOD COAGULATION.

A. E. Wright (*Proc. Roy. Soc.*, 1894 ; *J. Chem. Soc.*, 1895). The method used consisted in determining, by means of samples of blood withdrawn into capillary tubes, the alterations in the coagulability of the blood by altering the gaseous intake of living animals (dogs and rabbits). Increase of carbon dioxide increases the rate of coagulation. The same is true for human blood in cases of hemophilia. Diminution of the carbon dioxide decreases the coagulability to the original normal. Decrease and increase of oxygen cause respectively decrease and increase of coagulability.

---

CHANGES IN LIVER CELLS.

T. L. Brunton and S. Delépine (*Proc. Roy. Soc.*, 1894 ; *J. Chem. Soc.*, 1895). Various drugs were given to rabbits and the effect on the liver cells noted. The compounds studied may be subdivided into three groups : (1) stimulating or excito-secretory, with pilocarpine for a type ; (2) neutral ; and (3) depresso-secretory, with atropine for a type. To the first group belong toluene, benzene, sodium iodide, pilocarpine, chrysophanic acid, ammonium chloride, methatolylenediamine and nitric acid ; to the second, aniline and phenol ; to the third, phenol, atropine and ammonia. The following caused marked increase in glycogen : sodium iodide, methatolylenediamine, chrysophanic acid, toluene (?), ammonium chloride (?). The following did not do so, sometimes even causing a diminution of hepatic glycogen : nitric acid, pilocarpine, benzene and ammonium chloride (?). The following caused a marked diminution in the "free iron" in the liver : sodium iodide, toluene, methatolylenediamine. The following caused a diminution of the "free iron," but in a less marked degree : ammonium chloride, nitric acid, pilocarpine, benzene (in the fed liver) ; in the fasting liver benzene causes a doubtful increase of iron. Ammonia causes a diminution of glycogen and an increase of iron. Atropine causes a slight diminution of glycogen and little change in the iron.

---

ALBUMINURIA.

F. D. Boyd (Rep. of Lab. R. C. P., Edin., 1894 ; *J. Chem. Soc.*, 1895). The total proteïds were estimated by boiling, the albumin by boiling after removal of the globulin by half saturation with ammonium sulphate, the globulin by difference.

Both proteïds are generally present; the proportion varies so much that it is not possible to diagnose the variety of kidney disease present by means of it. Even in amyloid degeneration the globulin may not be in excess. In the albuminuria of pregnancy the globulin is present in larger amounts than in other forms of albuminuria. In the albuminuria of heart disease the globulin is usually more abundant than in chronic interstitial nephritis. In acute nephritis, without hematuria, the two proteïds are about equal; but when blood is present, the globulin is proportionally more abundant.

Four cases of Bright's disease are recorded in which serum albumin was present, but serum globulin absent, or present only in the merest traces; and one case where globulin and albumoses were present, but albumin absent. In no case did the proteïd quotient bear any relation to that of the blood. The amount of proteïd is often greater than that found in transudations. From these considerations the presence of proteïd in the urine is considered to be due to secretion rather than transudation, and there is some evidence that the lower the state of nutrition of the renal epithelium, the greater is the amount of globulin allowed to pass.

The urine secreted by the Malpighian tufts is by many physiologists regarded as albuminous. The albumin is considered to be reabsorbed as the urine passes along the renal tubules in the normal condition. Posner took pieces of the kidney and plunged them into boiling water for two or three minutes; sections were then prepared. Where albuminuria had been present the microscope revealed coagulated masses of proteïd in the capsule chambers and tubules, but when the healthy kidney, either of man or animals, was examined, no such evidence of albumin was ever found.

